# Virological and immunological outcome of treatment interruption in HIV-1-infected subjects vaccinated with MVA-B

**DOI:** 10.1371/journal.pone.0184929

**Published:** 2017-09-27

**Authors:** Miriam Rosás-Umbert, Beatriz Mothe, Marc Noguera-Julian, Rocío Bellido, Maria C. Puertas, Jorge Carrillo, C. Rodriguez, Núria Perez-Alvarez, Patricia Cobarsí, Carmen E. Gomez, Mariano Esteban, Jose Luis Jímenez, Felipe García, Julià Blanco, Javier Martinez-Picado, Roger Paredes, Christian Brander

**Affiliations:** 1 IrsiCaixa AIDS Research Institute—HIVACAT, Hospital Germans Trias i Pujol, Badalona, Spain; 2 Universitat Autònoma de Barcelona, Barcelona, Spain; 3 “Lluita contra la SIDA” Foundation, Hospital Germans Trias i Pujol, Badalona, Spain; 4 University of VIC and Central Catalonia, Vic, Spain; 5 Universitat Politecnica de Catalunya, Barcelona, Spain; 6 Centro Nacional de Biotecnología, CSIC, Madrid, Spain; 7 Hospital Gregorio Marañón, Madrid, Spain; 8 Hospital Clinic–HIVACAT, IDIBAPS, University of Barcelona, Barcelona, Spain; 9 Health Sciences Research Institute Germans Trias i Pujol, IGTP, Badalona, Spain; 10 Institució Catalana de Recerca i Estudis Avançats (ICREA), Barcelona, Spain; Rush University, UNITED STATES

## Abstract

The most relevant endpoint in therapeutic HIV vaccination is the assessment of time to viral rebound or duration of sustained control of low-level viremia upon cART treatment cessation. Structured treatment interruptions (STI) are however not without risk to the patient and reliable predictors of viral rebound/control after therapeutic HIV-1 vaccination are urgently needed to ensure patient safety and guide therapeutic vaccine development. Here, we integrated immunological and virological parameters together with viral rebound dynamics after STI in a phase I therapeutic vaccine trial of a polyvalent MVA-B vaccine candidate to define predictors of viral control. Clinical parameters, proviral DNA, host HLA genetics and measures of humoral and cellular immunity were evaluated. A sieve effect analysis was conducted comparing pre-treatment viral sequences to breakthrough viruses after STI. Our results show that a reduced proviral HIV-1 DNA at study entry was independently associated with two virological parameters, delayed HIV-1 RNA rebound (p = 0.029) and lower peak viremia after treatment cessation (p = 0.019). Reduced peak viremia was also positively correlated with a decreased number of HLA class I allele associated polymorphisms in Gag sequences in the rebounding virus population (p = 0.012). Our findings suggest that proviral DNA levels and the number of HLA-associated Gag polymorphisms may have an impact on the clinical outcome of STI. Incorporation of these parameters in future therapeutic vaccine trials may guide refined immunogen design and help conduct safer STI approaches.

## Introduction

Effective treatments for human immunodeficiency virus (HIV) infection exist and combination antiretroviral therapy (cART) has resulted in a dramatic decrease in morbidity and mortality. However, cART poses enormous challenges on global implementation and is not free of side effects [1][2][3][4]. Since HIV forms latent viral reservoirs from which the virus reactivates and replicates when treatment is interrupted, cART is a non-curative life-long treatment. Therapeutic vaccination in infected individuals aims to boost adaptive immunity against HIV and help to maintain viral replication at undetectable or low-levels in the absence of cART. The safe development of such strategies is complicated by the lack of well-defined parameters of HIV immune control and the uncertainties regarding most suitable endpoints in clinical vaccine trials. While results from cross-sectional cohorts of natural HIV infection point to various immune markers that are associated with viral load, no robust immune parameters have been identified that could serve as reliable predictors of viral control in patients receiving therapeutic vaccines and interrupting antiretroviral treatment [5][6][7][8].

Past therapeutic vaccine trials have oftentimes included structured treatment interruptions (STI) to assess the efficacy of tested vaccines and used control of viral rebound and/or prevention of CD4 T-cell decay upon treatment cessation as the primary trial endpoint [9][10][11]. However, STI is not free of risk to the health of infected individuals [12]. Therefore, it is generally only considered in well-controlled clinical trials that exclude patients with low CD4 counts, limit the duration of treatment cessation and use very stringent immune and virological criteria for treatment resumption after vaccine failure. A possibly less harmful approach to STI may be the so-called “monitored anti-retroviral pause” (MAP) where treatment is re-started at a pre-set (low) level of viral replication instead of after a pre-defined period off treatment [13]. In either way though, better predictors of viral rebound during MAP/STI are urgently needed to reduce the risk of conducting unyielding STI and to adjust trial design to maximize vaccination outcome.

Here, we tested such potential predictors of vaccine outcome in a recently completed therapeutic vaccine trial, referred to as RISVAC03. This trial was a double-blinded phase I clinical trial that assessed the safety and immunogenicity of an MVA-B candidate vaccine given alone or in combination with disulfiram in chronically infected, cART treated individuals who underwent STI post-vaccination [14][15][16]. In the present work, we sought to integrate host and vaccine-induced virological and immune parameters in order to study possible vaccine-exerted effects on rebounding virus, and to define correlates of viral rebound dynamics after STI.

## Material and methods

The study was approved by the Ethical Comitee of Hospital Clinic de Barcelona and the trial was registered at Clinicaltrials.gov number: NCT01571466.

### Patients and samples

The RISVAC03 study was a phase I double blinded, placebo-controlled therapeutic vaccine trial using an MVA vector expressing HIV-1 antigens from clade B (Bx08 gp120 and IIIB gag/pol/nef) with or without a drug to reactivate latent HIV (disulfiram) in successfully cART-treated, chronically HIV-positive individuals [14]. Of the 30 volunteers that participated in the study, 28 underwent an STI after completing full vaccination regimens consisting of three MVA-B vaccinations given intramuscularly [14]. Cryopreserved peripheral blood mononuclear cells (PBMC), plasma and serum samples were stored for immunological and virological studies. Two patients were excluded from the present analysis; one because of consent withdrawal before vaccination and a second one who did not interrupt ART after vaccination. The 28 participants consisted of 19 subjects in the vaccine arm and 9 in the placebo arm. Samples from pre-treatment time points were available from 13 individuals (11 in vaccine arm, 2 in placebo) and were used for plasma virus sequencing and sieve effect analyses. All individuals had started antiretroviral treatment 6 months or later after infection. High resolution HLA class I typing was performed by DNA sequence-based typing (SBT) and used for an assessment of escape mutations using described HLA footprint data and optimally defined CTL epitope lists as described [17] [18] [19].

### IFNg ELISPOT assay

PBMC were thawed and rested for 5 hours at 37°C before plating 100,000 live cells per well in IFN-γ ELISPOT 96-well polyvinylidene plates (Millipore). PBMC were stimulated with 42 pools of up to 23 peptides as described [14], discriminating HIV proteins that were, or were not, covered by the immunogen sequence contained in MVA-B. Briefly, IFN-γ ELISPOT responses were assessed using 15mer overlapping peptides combined into 25 pools covering the immunogen sequence (Env gp120 n = 6 pools, Gag n = 6, Pol n = 11 and Nef n = 2, referred to as “IN pools”) as well as the rest of the HIV-1 protein sequences not covered by MVA-B (17 pools: Gag p15 n = 1 pool, Pol int = 3, Vif n = 2, Vpr n = 1, Tat n = 1, Rev n = 1; Vpu n = 1, Nef n = 1 and Env gp41 n = 5, “OUT pools”). Un-stimulated cells served as a negative control and phytohemagglutinin (PHA 1μg/ml) stimulation was used as a positive control. Epitope specific, HLA-class I restricted CD8+ T cell responses were measured by determining the targeted optimal epitope based on the subjects HLA class I type, the 15mer reactivity data from previous Elispot screens and the described list of optimal CTL epitopes at the Los Alamos HIV Immunology database [17].

### Antibody detection

Plasma levels of anti- HIV native Env-specific IgG antibodies were determined by flow cytometry using MOLT cells expressing native functional Env glycoprotein (from isolates HIV-1_NL4.3_ and HIV-1_BaL_) or lacking Env expression as previously described [20] [21]. IgG binding Ab were quantified using a PE-F(ab)2 Goat anti-human IgG Fc specific (Jackson Immunoresearch) as secondary antibody and the signal expressed as the mean fluorescence intensity in the living MOLT cell gate.

### Reservoir and residual viremia determinations

Proviral HIV-1 DNA levels in purified CD4^+^ T-cell fractions were determined to assess the size of the viral reservoir over time. CD4^+^ T-cell lysates were used to measure the housekeeping gene RPP30 and total cell-associated HIV-1 DNA by quantitative droplet digital PCR (ddPCR, BioRad) in samples drawn before and after vaccination as well as at 2 and 12 weeks after treatment interruption, respectively [22]. Residual plasma viremia was quantified using Cobas® Ampliprep/Cobas® TaqMan® HIV-1 Test v2.0 (Roche), after ultracentrifugation of up to 14mL of plasma, in samples from visits at 2 and 12 weeks after treatment discontinuation. For the present study, the detection limit was 0.4–0.8 copies/mL.

### Sieve analysis

Ultra-deep sequencing of the HIV-1 gag gene was done using Illumina® NexteraXT protocol and MiSeq platform with 300 bp paired-end sequencing length [23] [24]. Raw sequence data was processed with Trimmomatic [25] to filter out low quality reads and trim adapter sequences. Good quality sequences were aligned against the HXB2R reference (genbank ID: K03455) using the bwa mem algorithm [26]. Mean depth of coverage of the resulting alignments was approximately 15.000. Amino acid variant calling was performed using a codon-level approach with an in-house pipeline. Amino acid variants were flagged if associated with HLA footprints specific for the patient’s HLA class I genotype. QuasiRecomb [27] software was used to obtain nucleotide positional entropy values and to reconstruct full gag haplotype sequences. Mean entropy values were calculated for Gag epitopes coordinates and the 4 most predominant haplotypes were used for analysis by MUSCLE multiple alignment with HXB2R and TN93+G+o nucleotidic distance calculation within the MEGA software [28].

### Statistical analysis

Quantitative data for longitudinal determinations were compared by paired Wilcoxon paired test. Comparisons between vaccinees and placebo recipients were analyzed using Mann-Whitney U test. Correlations were performed using the Spearman rank test. P-values <0.05 were considered to be statistically significant. GraphPad Prism version 4.0c (San Diego, CA) was used.

To define host and vaccine-induced virological and immune parameters that predict time to viral rebound (defined as weeks until there is detectable plasma viral load (>50 copies/mL)) after treatment interruption and the rebounding peak of viral load, we used two adjusted linear regression models. In both cases we included as independent variables: presence/absence of protective HLA class I alleles [29], MVA-B vaccination, magnitude of HIV-1 specific T cell responses, disulfiram administration, viral adaptation at CTL epitopes, pre-cART plasma VL, CD4+ T-cell counts and levels of proviral HIV-1 DNA. Linear regression models were adjusted using SPSS software Version 15.0 (SPSS Inc., Chicago, IL), and a p-value of less than 0.05 was considered significant.

## Results

### Delayed viral rebound in MVA-B vaccinees and increased cellular and humoral specific HIV immune responses after treatment interruption

**Fig 1:**
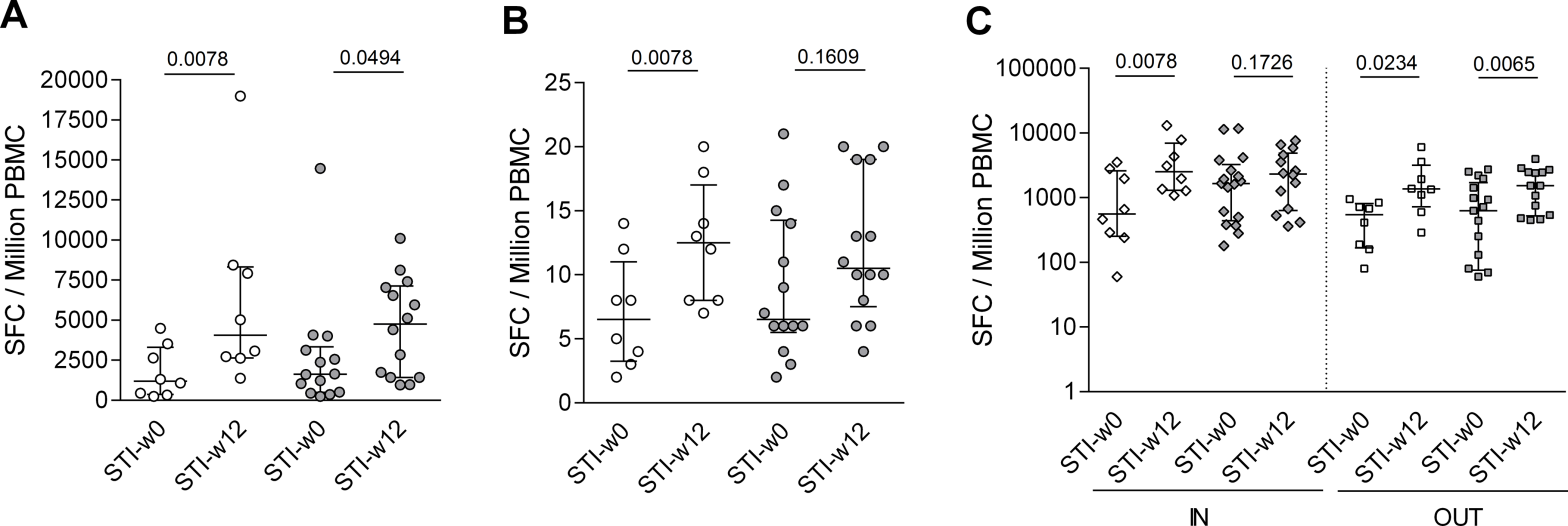
Increased cellular immune responses to HIV after treatment interruption. Magnitude (A) and breadth (B) of T cell responses to the entire HIV-1 proteome at start (STI-w0) and after w12 (STI-w12) of structured treatment in interruption (STI) is shown for placebo recipients (white) and the vaccinated group (grey). Median and interquartile range and p-values (Wilcoxon paired test) are shown. In (C), responses are divided into responses to regions of HIV that are covered (IN) or are not covered (OUT) by the MVA-B vaccine immunogen sequence.

The recently reported immunological and virological outcome MVA-B based therapeutic vaccination in the trial RISVAC03 demonstrated increased Gag-specific T cell responses in vaccinees compared to placebo controls [14]. The data also showed a modest but statistically significant delay in viral rebound in the vaccinees, particularly in vaccinated individuals not receiving disulfiram (p = 0.01) [14]. Here, we show that, in contrast to the modest elevation in magnitude of immune responses seen upon vaccination and reported previously[14] [16], there was a major increase in breadth and total magnitude of HIV-1 specific T cell responses after treatment interruption. Total magnitude of HIV-1 responses increased from a median of 1,235 to 4,054 SFC/10^6^ PBMC in placebos (p = 0.0078, Wilcoxon paired test) and median of 1,577 SFC/10^6^ PBMC at STI start to 4,767 SFC/10^6^ PBMC at 12 weeks after STI (p = 0.049, Wilcoxon paired test, Fig 1A) in vaccinees. The breadth of responses increased as well in both groups from initially a median of 6.5 pools to 10.5 in the vaccinees (p = 0.1531, Wilcoxon paired test) and, more pronounced, to 12.5 in placebo (p = 0.0078, Wilcoxon paired test, Fig 1B). Of note, placebo recipients increased their T cell responses targeting the vaccine insert as well as the rest of the HIV proteome upon treatment interruption (p = 0.0078, p = 0.0234 respectively, Wilcoxon paired test, Fig 1C). In contrast, in vaccinees, responses to targets not covered by the vaccine insert were boosted (p = 0.0067, Wilcoxon paired test, Fig 1C) while responses to regions contained in the immunogen sequence did not further expand.

**Fig 2:**
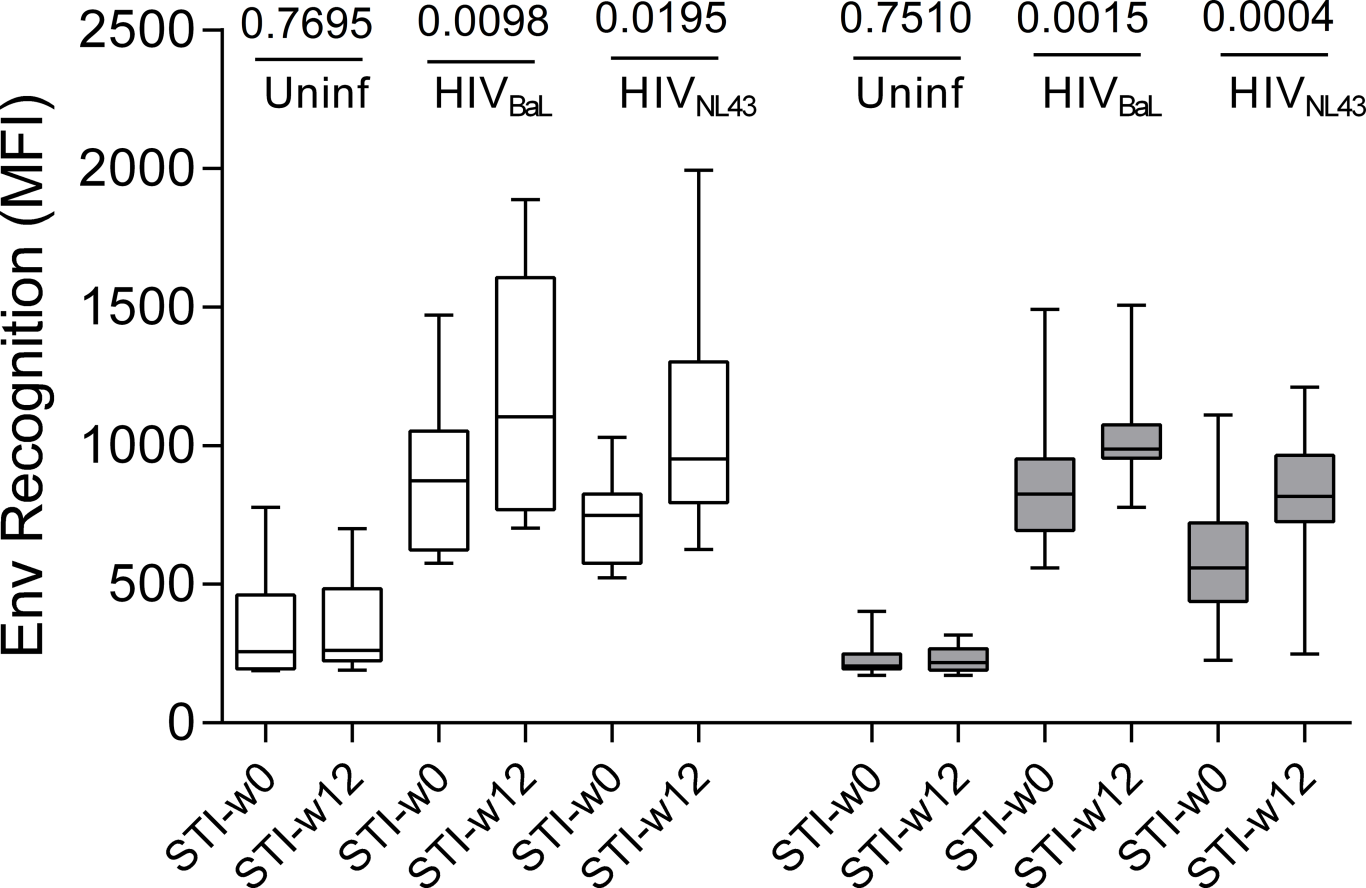
Structured treatment interruption (STI) increases levels of Env specific antibodies. Mean flourescence intensity (MFI) of stained MOLT cells expressing trimeric Env (from isolates HIV-1_NL4.3_ and HIV-1_BaL_) or lacking Env expression (uninfected) is show for plasma samples obtained at the start (STI-w0) or after 12 weeks (STI-w12) into STI in the placebo (white, n = 10) or the vaccinated (grey, n = 15) group. P-values for Wilcoxon paired test comparing w0-STI values to w12-STI are shown on top of the figure.

Concomitant with the increase in T-cell responses during treatment interruption, the levels of both, HIV_NL43_ and HIV_BaL_ Env-specific IgG increased significantly upon 12 weeks of STI compared either to baseline (pre-ART initiation) or STI-start. This was the case regardless whether all subjects were analyzed together (1.2 fold for HIV_BaL_ and 1.44 for HIV_NL43_, p<0.0001 for both, data not shown) or stratified by placebo (1.2 fold for HIV_BaL_, p = 0.0098; 1.3 times for HIV_NL43_, p = 0.0195) or vaccinees (1.3 fold for HIV_BaL_, p = 0.0015; 1.5 times for HIV_NL43_, p = 0.0004) (Fig 2). Together, these data demonstrate that rebound viral replication is positively associated with increased T- and B-cell responses to HIV, possibly due to antigen driven expansion of these cells.

### Viral reservoir is replenished after treatment interruption

**Fig 3:**
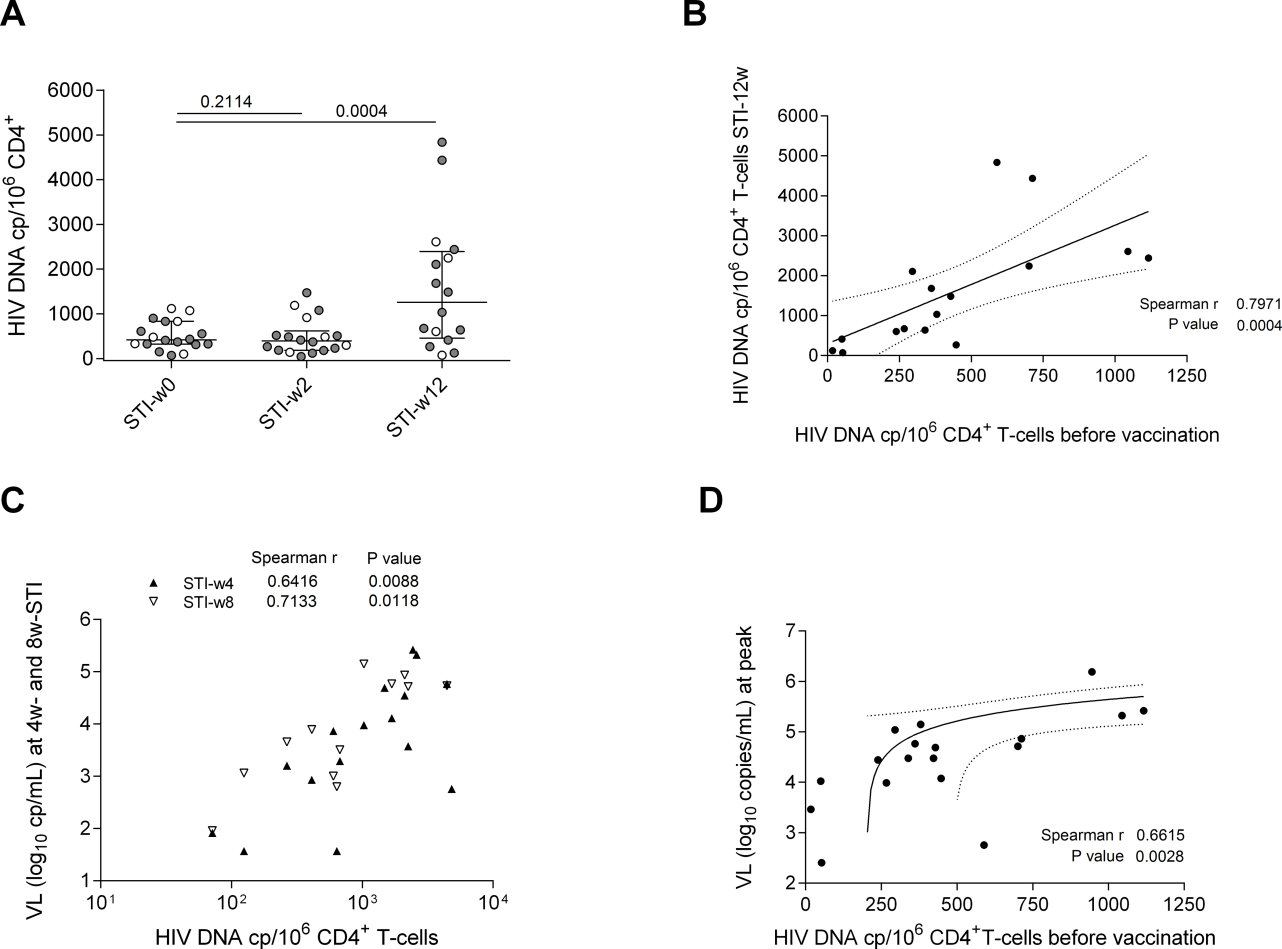
HIV-1 DNA copy numbers in CD4 cells before vaccination predicts extent of viral reservoir replenishment and plasma viral loads after structured treatment interruption (STI). (A) HIV DNA copy numbers in PBMC-derived, purified CD4+ T cells at start of STI (STI-w0), and 2 (STI-w2) or 12 (STI-w12) weeks after start of STI in placebo (white) and vaccinated individuals (grey). Median copy number (with interquartile range) is shown in all conditions (p-values Wilcoxon paired test). (B) Correlation between HIV DNA copy number in purified CD4+ T cells before any vaccination and after 12 weeks into STI (n = 16). Spearman correlation coefficient and p-value are shown. Linear regression line with 95% confidence intervals is represented. (C) Correlation between HIV DNA copy numbers per 10^6^ CD4 T cells at 12 weeks into STI and plasma viral loads (log_10_ copies/mL) 4 or 8 weeks after start of STI. Viral load at 4 weeks into STI (n = 16) is shown in white triangles and at 8 weeks (n = 12) in black triangles (r and p-value are shown for Spearman correlation). (D) Correlation between peak of viral load (log_10_ copies/mL) uring STI and HIV DNA copies detected before vaccination. Spearman correlation coefficient and p-value are shown. Linear regression line with 95% confidence intervals is represented.

To determine the effect of therapeutic vaccination on reservoir size and to monitor the extent of a possible replenishment of the viral reservoir upon STI, proviral HIV-1 DNA levels were determined in purified CD4^+^ T-cell fractions from 18 individuals (13 vaccines and 5 placebo) for whom sufficient PBMC material was available. There was no increase of proviral DNA at week 2 after STI although half of the patients had already experienced a detectable rebound in plasma viral load (median pVL 456 HIV RNA copies/mL, IQR: 192–1,515; assessed in 14/28 individuals that underwent STI, including 6 placebo and 8 vaccinees p = 0.2114) (Fig 3A). However, the proviral DNA increased in all of the 18 tested subjects by week 12 after STI with a median 3.1 fold increase (median 416 HIV DNA copies per million CD4^+^ T-cells to 1,257 p = 0.0004) (Fig 3A). Of note, proviral DNA levels in CD4^+^ T-cells after 12 weeks of STI correlated strongly with proviral DNA levels before vaccination (Fig 3B, p = 0.0002, r = 0.79). Furthermore, plasma viral load measured after 4, 8 and 12 weeks into STI showed a positive correlation with levels of proviral DNA at 12 weeks of STI (n = 16 at STI-w4 Spearman r = 0.6416 p = 0.0074, n = 12 at STI-w8 Spearman r = 0.7133 p = 0.0092 and n = 8 at STI-w18 Spearman r = 0.9048, p = 0.0020) (Fig 3C). Proviral DNA levels in CD4+ T cells before vaccination were also associated with peak of viral load during viral rebound (n = 18, Spearman r = 0.6615 p = 0.0028) (Fig 3D). Although there was no difference in the increase in proviral DNA levels between placebo recipients and vaccinated individuals, these data indicate that rebounding viremia precedes reservoir reseeding. The data also show that pre-vaccination proviral DNA predicts the extent of reseeding of viral reservoir reseeding in CD4^+^ T cells and peak viremia after treatment interruption.

### No evidence for a strong immune selection pressure on rebounding viral population during STI

**Fig 4:**
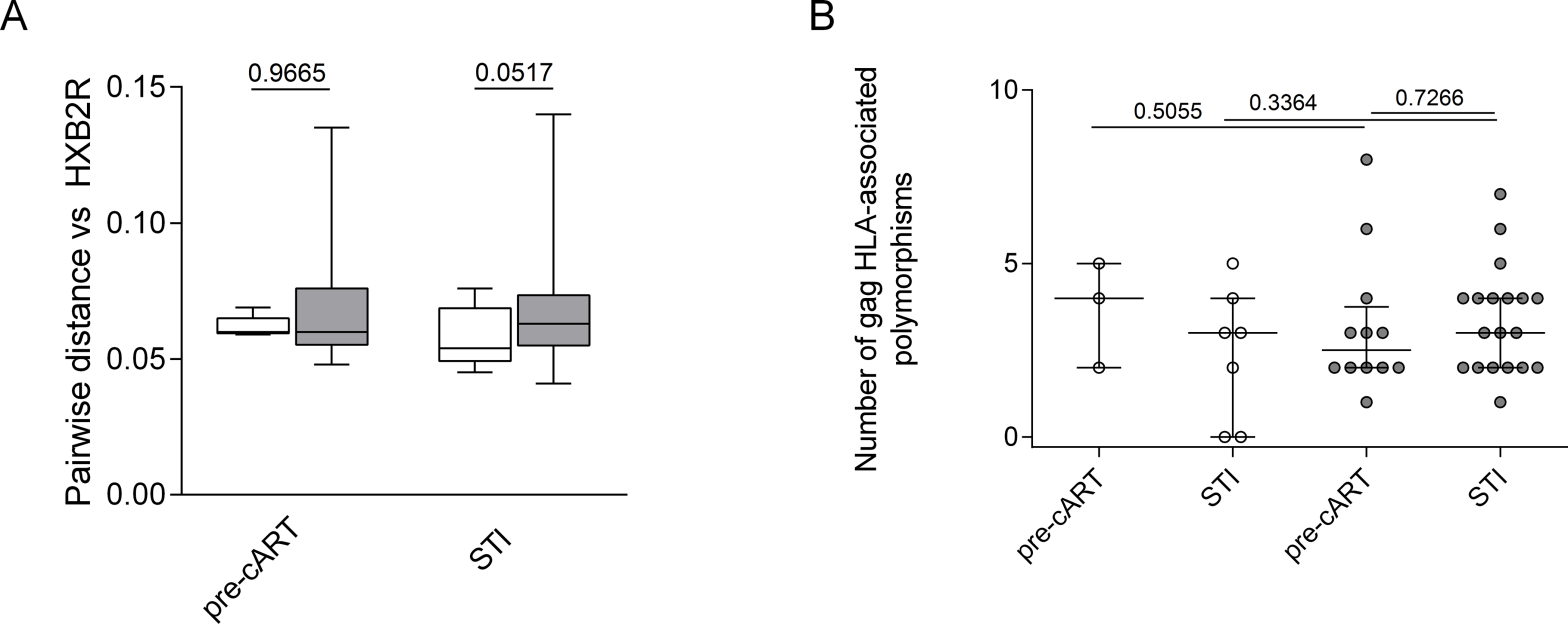
No evidence of immune selection pressure in rebounding virus after therapeutic MVA-B vaccination. (A) Pairwise distances to the reference sequence HXB2 is shown for samples obtained before ART initiation and after 2–12 weeks of treatment interruption. P-value for Mann Whitney test between defined groups (placebo and vaccinated) is shown. (B) The number of HLA-associated polymorphisms in Gag is shown for sequences obtained before cART (n = 3 placebo, n = 12 vaccines) and during viral rebound after STI (n = 7 placebo, n = 19 vaccines). P-value is shown for Wilcoxon paired test when comparing between pre-cART and STI data and p-value for Mann Whitney test is shown to compare groups.

To determine whether the vaccine-induced immune response exerted a noticeable selection pressure on rebounding virus, we conducted a sieve effect analysis on the expanding viral populations after STI. Deep sequencing of viral gag RNA was performed in plasma samples obtained pre-cART treatment start (n = 12 individuals (2 placebos, 10 vaccinated subjects with a total of 29 (5 + 24) sequences) and at 2 or 12 weeks after STI when viremic plasma samples were available (n = 26 individuals, 8 placebos and 18 vaccinated subjects with a total of 105 (24 +81) sequences). The phylogenetic distance to a reference sequence (HXB2) and entropy values were determined. The analysis showed no difference of pairwise distances of Gag sequences to the reference sequence HXB2 between placebo and vaccinated group, neither for time points before starting any cART or during STI (Fig 4A). Similarly, there were no differences in the entropy values between placebo recipients and vaccinees and between baseline sequences (before any cART) and after STI (data not shown). Thus, even though vaccination with MVA-B was immunogenic, these responses did not exert a measurable immune selection pressure on the rebounding viruses.

To further assess whether the rebounding virus showed signs of successful CTL escape on a single epitope level, the number of HLA associated immune escape mutations were determined based on the individual’s HLA class I genotype and compared between samples pre-cART and after STI. There was no increase in the number of HLA class I allele-specific polymorphisms in the *Gag* sequences of the rebounding virus when compared to samples drawn pre-cART initiation (Wilcoxon paired test p = 0.3833 for placebo, p = 0.5469 for vaccinated group) and no differences between placebo and vaccines recipients for either time point (Mann-Whitney test p = 0.3685 for samples before cART, p = 0.2058 for samples during STI) (Fig 4B). In fact, 78% of Gag escape polymorphisms (35 out of the 45 detected in 13 subjects available for analysis) were already present in the viral sequences before starting cART while only 6.5% of polymorphisms (n = 3) appeared after STI and 15.5% (n = 7) were lost in between the two time points. In three individuals with available PBMC samples, we were able to relate the occurrence of mutations in T-cell epitopes with a reduced stimulation of epitope-specific T cells, but these analyses were too limited to draw broader conclusions (data not shown).

### Proviral DNA is associated with time to rebound and peak of viral load after treatment interruption

To determine whether individual or combinations of the above markers had the power to predict viral load kinetics and plasma viral load set point (i.e. pVL at STI-w12), we integrated all data generated from all subjects in a linear regression model that included: presence of protective HLA class I alleles (B*57, B*27 or B*51), receipt of MVA-B vaccination/placebo, total magnitude of HIV-1 specific T-cell responses, disulfiram administration, number of HLA footprints in Gag, pre-cART pVL, CD4 cell counts and levels of proviral HIV-1 DNA before STI. The results show that the only variable that was independently associated with delayed time to rebound (measured as weeks until there is a detectable plasma viral load) was proviral HIV-1 DNA before any vaccination (p = 0.029) (Table 1). In this model, an increase of 100 copies in the number of proviral HIV-1 DNA/10^6^ CD4 T cells decreases the time to rebound by a mean of 2 days. Taking in account the size of our dataset and considering 95% confidence levels, there was no multivariate model emerging that could predict the time of viral rebound. Nevertheless, there was a model that predicted peak of viremia after treatment interruption and which included proviral pre-vaccination HIV-1 DNA (p = 0.019) and the number of HLA-associated polymorphisms in Gag (p = 0.012) (Table 2). In this model, one additional HLA-associated Gag escape mutation increases the mean peak VL by 114,290 copies/ml, while an increase of 100 copies of HIV DNA/10^6^ CD4 augment the mean viral load by 55,900 copies/ml.

**Table 1:** Regression model for "*time to rebound*”.

	Coeff	P-value	95%CI-lower	95%CI-upper
Vaccine /placebo arm	1,228	0,103	-0,265	2,721
Disulfiram/ no disulfiram administration	-0,075	0,919	-1,579	1,429
HLA protective / non- protective	-0,429	0,551	-1,888	1,031
Viral Load pre cART	0,000	0,725	0,000	0,000
CD4 w0-STI	-0,001	0,360	-0,003	0,001
Magnitude w0-STI (SFC/10^6^ PMBC)	0,000	0,762	0,000	0,000
Magnitude w12-STI (SFC/10^6^ PMBC)	0,000	0,572	0,000	0,000
Proviral DNA before vaccination (HIV DNA copies/10^6^ CD4 cells)	-0,003	0,029	-0,005	0,000
Residual Viremia baseline	0,094	0,650	-0,338	0,526
Number of gag HLA-associated escape mutations	-0,926	0,751	-6,887	5,036

Regression model for the dependent variable "*time to rebound*” (weeks until detectable plasma viremia) was performed on all the individuals undergoing STI (n = 28) and including the following covariates: harboring protective HLA alleles (B*27, B*57 and B*51 (n = 14)), MVA-B vaccination (n = 19), magnitude of HIV-1 specific T cell responses at STI-start (n = 26) and after 12 weeks into STI (n = 24), disulfiram administration (n = 12), CTL virus adaptation (number of total Gag polymorphism and number of HLA-associated escape mutations in Gag) (n = 26), pre-HAART pVL (n = 25), CD4 cell counts (n = 28), residual viremia (n = 18) and levels of proviral HIV-1 DNA at the beginning of the study (n = 18). Coefficients and p-values are shown.

**Table 2:** Univariate and multivariate regression model for "*peak of viral load at rebound*”.

	Univariate model				Multivariate model			
	Coeff	P-value	95%CI-lower	95%CI-upper	Coeff	P-value	95%CI-lower	95%CI-upper
Vaccine/placebo arm	-6902.047	0.955	-258614.004	244809.91				
Disulfiram/ no disulfiram administration	166864.439	0.150	-64263.410	397992.29				
HLA protective/ non- protective	78599.429	0.494	-154381.726	311580.58				
Viral Load pre cART	-0.016	0.955	-0.594	0.564				
CD4 w0-STI	-107.151	0.555	-475.123	260.822				
Magnitude w0-STI	-0.044	0.998	-35.594	35.506				
Magnitude w12-STI	-4.746	0.783	-40.192	30.700				
Proviral DNA before vaccination	551.160	0.033	51.021	1051.298	558.952	0.019	28853.106	199727.531
Residual Viremia baseline	6247.651	0.880	-80093.639	92588.941				
Number of gag HLA-associated escape mutations	63348.616	0.080	-8278.874	134976.107	114290.319	0.012	107.529	1010.375
Time to rebound	-45543.989	0.142	-107383.229	16295.250				

Regression model for the dependent variable "*peak of viral load at rebound*” was performed including the following covariates: harboring protective HLA alleles (B*27, B*57 and B*51 (n = 14)), MVA-B vaccination (n = 19), magnitude of HIV-1 specific T cell responses at STI-start (n = 26) and after 12 weeks into STI (n = 24), disulfiram administration (n = 12), CTL virus adaptation (number of total Gag polymorphism and number of HLA-associated escape mutations in Gag) (n = 26), pre-HAART pVL (n = 25), CD4 cell counts (n = 28), residual viremia (n = 18) and levels of proviral HIV-1 DNA at the beginning of the study (n = 18). Coefficients and p-values are shown for a univariate and multivariate model.

## Discussion

Predictors and immune markers for successful outcome of therapeutic HIV vaccination have not been defined, partially due to the lack of strong clinical effects of such therapeutic immune interventions to date. A detailed analysis of the recently completed RISVAC03 trial provided the opportunity to test some virological and immune parameters that could predict the clinically modest, but statistically significant delay in viral rebound seen in vaccinees in this trial. In RISVAC03, all but two individuals who underwent treatment interruption rebounded plasma viral load and had to restart cART [14]. Neither the vaccine-induced increase in Gag-specific T cell responses nor CD4+ T-cell counts before treatment interruption were associated with the time to rebound or levels of peak viral load during STI. We here added markers of virus-specific T- and B-cell immunity, phylogenetic studies of the viral population pre/post STI and detailed reservoir determinations to the analysis of viral rebound kinetics.

CTL and B cell responses increased rapidly in breadth and total magnitude during the treatment interruption in both, vaccine and placebo arms, which is likely explained by the resurgence of viral antigen driving these responses [9][30][31][32]. Since the vaccine-induced responses have been shown previously to be of an effector memory phenotype [16] and to include non-functional CD8+ T cells with elevated PD-1 expansion [15], the T cell responses expanded by rebounding virus may be pre-existing, previously ineffective T cells, unable to control viral replication. A failure to expand functionally active and non-exhausted T cells in the RISVAC03 trial may also explain the failure to observe a significant selection pressure on the rebounding virus [33][34][35]. However, it is also important to note that CTL epitope mutations were largely present in samples from pre cART already and that the source of rebounding virus was already highly adapted viral species. This is in line with viral adaptation to cellular immune responses during primary infection and reflective of a population treated during chronic phase of HIV infection [36][37][38][39][40][41][42]. However, sieve effect analyses in the therapeutic setting may be different from preventive vaccine studies. For instance, a post-hoc analysis of the STEP vaccine trial [33][43][35] showed that breakthrough infections in vaccinated individuals harbored viral sequences that had a significantly greater phylogenetic distance to the vaccine immunogen sequence when compared to viral sequence in individuals that had received placebo. Similar sieve effect analyses have also been conducted, among others, in the RV144 and in the therapeutic DC-TRN vaccine trials [11][44][45] and provide some evidence of vaccine-induced immune selection pressure.

In the present study, half of the patients rebounded plasma viral load rapidly and had detectable plasma viremia after 2 weeks of STI. However, levels of proviral reservoir did not show an increase at that point in either the vaccines or the placebo, suggesting that the viral reservoir, at least in the peripheral blood compartment, has slower kinetics of replenishment than kinetics of viral load rebound detected in plasma. This may offer a window of opportunity to prevent complete reseeding of reservoir, at least in individuals treated in chronic stage, if viral load measurements are conducted with short intervals and cART is resumed immediately after viral rebound is detected. This argues in favor of monitored antiretroviral pause (MAP) that reintroduces ART as soon as viral rebound occurs rather than STI strategy when assessing the efficacy of an HIV therapeutic vaccine [46].

Finally, we analyzed the variables associated with viral rebound at STI week 12 using linear regression models and identified baseline proviral DNA before vaccination as a predictor of the time to viral rebound and peak of viral load after treatment interruption. Similar observations have been made in a study by Li who used a therapeutic rAd5 HIV-1 Gag vaccine [47] [48] and in studies that assessed the outcome of treatment interruptions in people with acute HIV infection [49] [50], showing higher levels of HIV DNA levels to be associated with more rapid viral rebound. However, a meta-analysis of six AIDS Clinical Trials Group STI studies [51] suggests that additional biological factors participate in a complex relationship that define the outcome of STI studies. The participation of additional factors aside from reservoir size is also highlighted by recent clinical examples where in the absence of a targeted immune therapy and even in the absence of undetectable viral reservoirs, viral rebound can occur [52] [53]. Combinational approaches, including ‘kick and kill’ strategies may thus be necessary for a functional cure, where latency reversal agents (LRA), neutralizing antibodies or innate immune stimulators would be administered in combination with therapeutic T cell vaccines to boost the host’s antiviral immunity [54]. This is supported by recent data in SIV-infected rhesus monkeys where a therapeutic vaccination together with toll-like receptor 7 (TLR7) stimulation improved virological control and delayed viral rebound following ART discontinuation [55].

In conclusion, our data provide insights into factors of viral rebound in a therapeutic vaccine trial that showed a statistically significant delay in viral rebound kinetics in vaccinated individuals. Although the data will need to be validated in larger clinical trials, they may further our understanding of potential mechanisms of vaccine control and help improve safety of future clinical trials. Such analyses would further benefit from the inclusion of more immunogenic vaccines as the signals of HIV escape seen here may be more pronounced and may offer the opportunity to identify novel immune correlates of sustained viral control.

### Members of the RISVAC-03 study group

Irsicaixa AIDS Research Institute-HIVACAT, Hospital Germans Trias i Pujol, Badalona, Spain: Beatriz Mothe, Patricia Cobarsi, Miriam Rosás-Umbert, María C. Puertas, Jorge Carrillo, Julià Blanco, Javier Martinez-Picado, Bonaventura Clotet and Christian Brander.

Hospital Clinic-HIVACAT, IDIBAPS, University of Barcelona, Spain: Nuria Climent, Montserrat Plana, Carmen Alvarez, Sonsoles Sánchez, Agathe León, Judit Pich, Joan Albert Arnaiz, Lorna Leal, Berta Torres, Constanza Lucero, Alberto C. Guardo, Jose M. Gatell and Felipe García.

Hospital Gregorio Marañón, Madrid, Spain: José Luis Jiménez, María Angeles Muñoz-Fernández and Juan Carlos López Bernaldo de Quirós.

Centro Nacional de Biotecnología, CSIC, Madrid, Spain: Mariano Esteban, Carmen Elena Gómez, Beatriz Perdiguero, Juan García-Arriaza, Victoria Cepeda and Carlos Oscar Sánchez-Sorzano.

Instituto de Salud Carlos III, Madrid, Spain: Nuria Gonzalez, José Alcamí and Laura Jiménez.

Instituto de Investigación Sanitaria—Fundación Jiménez Díaz, Madrid, Spain: José M. Benito and Norma Rallón.

Hospital Reina Sofía, Córdoba, Spain: José Peña.

## References

[1] Global AIDS Update 2016 | UNAIDS [Internet]. [cited 1 Mar 2017]. Available: http://www.unaids.org/en/resources/documents/2016/Global-AIDS-update-2016

[2] Crawford KW, Ripin DHB, Levin AD, Campbell JR, Flexner C, participants of Conference on Antiretroviral Drug Optimization. Optimising the manufacture, formulation, and dose of antiretroviral drugs for more cost-efficient delivery in resource-limited settings: a consensus statement. Lancet Infect Dis. 2012;12: 550–60. doi: 10.1016/S1473-3099(12)70134-2 22742638 10.1016/S1473-3099(12)70134-2

[3] Holmes CB, Coggin W, Jamieson D, Mihm H, Granich R, Savio P, et al Use of generic antiretroviral agents and cost savings in PEPFAR treatment programs. JAMA. 2010;304: 313–20. doi: 10.1001/jama.2010.993 20639565 10.1001/jama.2010.993

[4] Katlama C, Deeks SG, Autran B, Martinez-picado J, van Lunzen J, Rouzioux C, et al Barries to a Cure: New concepts in targeting and eradicating HIV-1 reservoirs. Lancet. 2013;381 doi: 10.1016/S0140-6736(13)60104-X.Barriers 10.1016/S0140-6736(13)60104-X PMC3815451 23541541

[5] Li JZ, Brumme ZL, Brumme CJ, Wang H, Spritzler J, Robertson MN, et al Factors associated with viral rebound in HIV-1-infected individuals enrolled in a therapeutic HIV-1 gag vaccine trial. J Infect Dis. 2011;203: 976–83. doi: 10.1093/infdis/jiq143 21402549 10.1093/infdis/jiq143 PMC3068025

[6] Papagno L, Alter G, Assoumou L, Murphy RL, Garcia F, Clotet B, et al Comprehensive analysis of virus-specific T-cells provides clues for the failure of therapeutic immunization with ALVAC-HIV vaccine. AIDS. 2011;25: 27–36. doi: 10.1097/QAD.0b013e328340fe55 21076273 10.1097/QAD.0b013e328340fe55

[7] Li JZ, Heisey A, Ahmed H, Wang H, Zheng L, Carrington M, et al Relationship of HIV reservoir characteristics with immune status and viral rebound kinetics in an HIV therapeutic vaccine study. AIDS. 2014;28: 2649–57. doi: 10.1097/QAD.0000000000000478 25254301 10.1097/QAD.0000000000000478 PMC4267919

[8] Hurst J, Hoffmann M, Pace M, Williams JP, Thornhill J, Hamlyn E, et al Immunological biomarkers predict HIV-1 viral rebound after treatment interruption. Nat Commun. Nature Publishing Group; 2015;6: 8495 doi: 10.1038/ncomms9495 26449164 10.1038/ncomms9495 PMC4633715

[9] Jacobson JM, Pat Bucy R, Spritzler J, Saag MS, Eron JJ, Coombs RW, et al Evidence that intermittent structured treatment interruption, but not immunization with ALVAC-HIV vCP1452, promotes host control of HIV replication: the results of AIDS Clinical Trials Group 5068. J Infect Dis. 2006;194: 623–32. doi: 10.1086/506364 16897661 10.1086/506364

[10] Autran B, Murphy RL, Costagliola D, Tubiana R, Clotet B, Gatell J, et al Greater viral rebound and reduced time to resume antiretroviral therapy after therapeutic immunization with the ALVAC-HIV vaccine (vCP1452). AIDS. 2008;22: 1313–22. doi: 10.1097/QAD.0b013e3282fdce94 18580611 10.1097/QAD.0b013e3282fdce94

[11] De Goede AL, van Deutekom HWM, Vrancken B, Schutten M, Allard SD, van Baalen C a, et al HIV-1 evolution in patients undergoing immunotherapy with Tat, Rev, and Nef expressing dendritic cells followed by treatment interruption. AIDS. 2013;27: 2679–89. doi: 10.1097/01.aids.0000433813.67662.92 24149085 10.1097/01.aids.0000433813.67662.92

[12] El-Sadr WM, Abrams D, Losso M, General De Agudos H-T, Ramos Mejia JM, Rappoport C. CD4+ Count–Guided Interruption of Antiretroviral Treatment. n engl j med. 2006;35522. 10.1056/NEJMoa062360 17135583

[13] Anderson JL, Mi Fromentin R, Corbelli GM, Østergaard L, Ross AL. Progress Towards an HIV Cure: Update from the 2014 International AIDS Society Symposium. 10.1089/aid.2014.0236 10.1089/aid.2014.0236 PMC4287112 25257573

[14] Mothe B, Climent N, Plana M, Rosas M, Jimenez JL, Munoz-Fernandez M a., et al Safety and immunogenicity of a modified vaccinia Ankara-based HIV-1 vaccine (MVA-B) in HIV-1-infected patients alone or in combination with a drug to reactivate latent HIV-1. J Antimicrob Chemother. 2015; 1–10. doi: 10.1093/jac/dku458 25724985 10.1093/jac/dkv046

[15] Rallón N, Mothe B, Lopez Bernaldo de Quiros JC, Plana M, Ligos, Montoya M, et al Balance between activation and regulation of HIV-specific CD8+ T-cell response after modified vaccinia Ankara B therapeutic vaccination. AIDS. 2016;30: 553–62. doi: 10.1097/QAD.0000000000000966 26558724 10.1097/QAD.0000000000000966

[16] Gómez CE, Perdiguero B, García-Arriaza J, Cepeda V, Sánchez-Sorzano CÓ, Mothe B, et al A Phase I Randomized Therapeutic MVA-B Vaccination Improves the Magnitude and Quality of the T Cell Immune Responses in HIV-1-Infected Subjects on HAART. PLoS One. 2015;10: e0141456 doi: 10.1371/journal.pone.0141456 26544853 10.1371/journal.pone.0141456 PMC4636254

[17] Llano A, Frahm N. I-A How to Optimally Define Optimal Cytotoxic T Lymphocyte Epitopes in HIV Infection? Mol Immunol. 2009; 3–24. doi: 10.1016/j.molimm.2008.12.01919193443

[18] Brumme ZL, John M, Carlson JM, Brumme CJ, Chan D, Brockman M a, et al HLA-associated immune escape pathways in HIV-1 subtype B Gag, Pol and Nef proteins. PLoS One. 2009;4: e6687 doi: 10.1371/journal.pone.0006687 19690614 10.1371/journal.pone.0006687 PMC2723923

[19] Carlson JM, Brumme CJ, Martin E, Listgarten J, Brockman M a, Le AQ, et al Correlates of protective cellular immunity revealed by analysis of population-level immune escape pathways in HIV-1. J Virol. 2012;86: 13202–16. doi: 10.1128/JVI.01998-12 23055555 10.1128/JVI.01998-12 PMC3503140

[20] Blanco J, Barretina J, Clotet B, Esté JA. R5 HIV gp120-mediated cellular contacts induce the death of single CCR5-expressing CD4 T cells by a gp41-dependent mechanism. J Leukoc Biol. 2004;76: 804–11. doi: 10.1189/jlb.0204100 15258189 10.1189/jlb.0204100

[21] Sánchez-Palomino S, Massanella M, Carrillo J, García A, García F, González N, et al A cell-to-cell HIV transfer assay identifies humoral responses with broad neutralization activity. Vaccine. 2011;29: 5250–5259. doi: 10.1016/j.vaccine.2011.05.016 21609746 10.1016/j.vaccine.2011.05.016

[22] Martínez-Bonet M, Puertas MC, Fortuny C, Ouchi D, Mellado MJ, Rojo P, et al Establishment and Replenishment of the Viral Reservoir in Perinatally HIV-1-infected Children Initiating Very Early Antiretroviral Therapy. Clin Infect Dis. 2015;61: 1169–1178. doi: 10.1093/cid/civ456 26063721 10.1093/cid/civ456 PMC4560905

[23] Bentley DR, Balasubramanian S, Swerdlow HP, Smith GP, Milton J, Brown CG, et al Accurate Whole Human Genome Sequencing using Reversible Terminator Chemistry. Nature. 2009;456: 53–59. doi: 10.1038/nature07517.Accurate 10.1038/nature07517 PMC2581791 18987734

[24] Syed F, Grunenwald H, Caruccio N. Optimized library preparation method for next-generation sequencing. Nat Methods, Publ online 01 Oct 2009; |. Nature Publishing Group; 2009;6 doi: 10.1038/NMETH.F.269

[25] Bolger AM, Lohse M, Usadel B. Trimmomatic: A flexible trimmer for Illumina sequence data. Bioinformatics. 2014;30: 2114–2120. doi: 10.1093/bioinformatics/btu170 24695404 10.1093/bioinformatics/btu170 PMC4103590

[26] Li H, Durbin R. Fast and accurate long-read alignment with Burrows-Wheeler transform. Bioinformatics. 2010;26: 589–595. doi: 10.1093/bioinformatics/btp698 20080505 10.1093/bioinformatics/btp698 PMC2828108

[27] Töpfer A, Zagordi O, Prabhakaran S, Roth V, Halperin E, Beerenwinkel N. Probabilistic inference of viral quasispecies subject to recombination. J Comput Biol. Mary Ann Liebert, Inc.; 2013;20: 113–23. doi: 10.1089/cmb.2012.0232 23383997 10.1089/cmb.2012.0232 PMC3576916

[28] Tamura K, Peterson D, Peterson N, Stecher G, Nei M, Kumar S. MEGA5: molecular evolutionary genetics analysis using maximum likelihood, evolutionary distance, and maximum parsimony methods. Mol Biol Evol. Oxford University Press; 2011;28: 2731–9. doi: 10.1093/molbev/msr121 21546353 10.1093/molbev/msr121 PMC3203626

[29] Goulder PJ, Walker BD. HIV and HLA class I: an evolving relationship. Immunity. Elsevier Inc.; 2012;37: 426–440. doi: 10.1016/j.immuni.2012.09.005 22999948 10.1016/j.immuni.2012.09.005 PMC3966573

[30] Daucher M, Price DA, Brenchley JM, Lamoreaux L, Metcalf JA, Rehm C, et al Virological outcome after structured interruption of antiretroviral therapy for human immunodeficiency virus infection is associated with the functional profile of virus-specific CD8+ T cells. J Virol. American Society for Microbiology (ASM); 2008;82: 4102–14. doi: 10.1128/JVI.02212-07 18234797 10.1128/JVI.02212-07 PMC2292997

[31] Thompson M, Heath SL, Sweeton B, Williams K, Cunningham P, Keele BF, et al. DNA / MVA Vaccination of HIV-1 Infected Participants with Viral Suppression on Antiretroviral Therapy, followed by Treatment Interruption: Elicitation of Immune Responses without Control of Re- Emergent Virus. 2016; 1–25. 10.1371/journal.pone.0163164 10.1371/journal.pone.0163164 PMC5053438 27711228

[32] Llano a, Carrillo J, Mothe B, Marfil S, García E, Ruiz L, et al Modulation of antibody secreting cells and neutralizing Ab activity in HIV infected individuals undergoing structured treatment interruptions. Retrovirology. 2012;9: P63 doi: 10.1186/1742-4690-9-S2-P63 10.1186/1479-5876-11-48 PMC3605223 23433486

[33] Rolland M, Tovanabutra S, deCamp AC, Frahm N, Gilbert PB, Sanders-Buell E, et al Genetic impact of vaccination on breakthrough HIV-1 sequences from the STEP trial. Nat Med. NIH Public Access; 2011;17: 366–71. doi: 10.1038/nm.2316 21358627 10.1038/nm.2316 PMC3053571

[34] Janes H, Frahm N, DeCamp A, Rolland M, Gabriel E, Wolfson J, et al MRKAD5 HIV-1 Gag/Pol/Nef vaccine-induced T-cell responses inadequately predict distance of breakthrough HIV-1 sequences to the vaccine or viral load. PLoS One. 2012;7 doi: 10.1371/journal.pone.0043396 22952672 10.1371/journal.pone.0043396 PMC3428369

[35] Edlefsen PT, Gilbert PB, Rolland M. Sieve analysis in HIV-1 vaccine efficacy trials. Curr Opin HIV AIDS. 2013;8: 432–6. doi: 10.1097/COH.0b013e328362db2b 23719202 10.1097/COH.0b013e328362db2b PMC3863593

[36] Price D a, Goulder PJ, Klenerman P, Sewell a K, Easterbrook PJ, Troop M, et al Positive selection of HIV-1 cytotoxic T lymphocyte escape variants during primary infection. Proc Natl Acad Sci U S A. 1997;94: 1890–5. doi: 10.1073/pnas.94.5.1890 9050875 10.1073/pnas.94.5.1890 PMC20013

[37] Liu Y, McNevin J, Cao J, Zhao H, Genowati I, Wong K, et al Selection on the human immunodeficiency virus type 1 proteome following primary infection. J Virol. 2006;80: 9519–29. doi: 10.1128/JVI.00575-06 16973556 10.1128/JVI.00575-06 PMC1617227

[38] Brumme ZL, Brumme CJ, Carlson J, Streeck H, John M, Eichbaum Q, et al Marked epitope- and allele-specific differences in rates of mutation in human immunodeficiency type 1 (HIV-1) Gag, Pol, and Nef cytotoxic T-lymphocyte epitopes in acute/early HIV-1 infection. J Virol. 2008;82: 9216–27. doi: 10.1128/JVI.01041-08 18614631 10.1128/JVI.01041-08 PMC2546878

[39] Fischer W, Ganusov V V., Giorgi EE, Hraber PT, Keele BF, Leitner T, et al Transmission of single HIV-1 genomes and dynamics of early immune escape revealed by ultra-deep sequencing. PLoS One. 2010;5 doi: 10.1371/journal.pone.0012303 20808830 10.1371/journal.pone.0012303 PMC2924888

[40] Henn MR, Boutwell CL, Charlebois P, Lennon NJ, Power K a., Macalalad AR, et al Whole genome deep sequencing of HIV-1 reveals the impact of early minor variants upon immune recognition during acute infection. PLoS Pathog. 2012;8 doi: 10.1371/journal.ppat.1002529 22412369 10.1371/journal.ppat.1002529 PMC3297584

[41] Martin E, Carlson JM, Le AQ, Chopera DR, McGovern R, Rahman M a, et al Early immune adaptation in HIV-1 revealed by population-level approaches. Retrovirology. 2014;11: 64 doi: 10.1186/s12977-014-0064-1 25212686 10.1186/s12977-014-0064-1 PMC4190299

[42] Deng K, Pertea M, Rongvaux A, Wang L, Durand CM, Ghiaur G, et al Broad CTL response is required to clear latent HIV-1 due to dominance of escape mutations. Nature. Nature Research; 2015;517: 381–385. doi: 10.1038/nature14053 25561180 10.1038/nature14053 PMC4406054

[43] Rolland M, Edlefsen PT, Larsen BB, Tovanabutra S, Sanders-Buell E, Hertz T, et al Increased HIV-1 vaccine efficacy against viruses with genetic signatures in Env V2. Nature. NIH Public Access; 2012;490: 417–20. doi: 10.1038/nature11519 22960785 10.1038/nature11519 PMC3551291

[44] Buchbinder SP, Mehrotra D V, Duerr A, Fitzgerald DW, Mogg R, Li D, et al Efficacy assessment of a cell-mediated immunity HIV-1 vaccine (the Step Study): a double-blind, randomised, placebo-controlled, test-of-concept trial. Lancet (London, England). NIH Public Access; 2008;372: 1881–93. doi: 10.1016/S0140-6736(08)61591-3 19012954 10.1016/S0140-6736(08)61591-3 PMC2721012

[45] Dommaraju K, Kijak G, Carlson JM, Larsen BB, Tovanabutra S, Geraghty DE, et al CD8 and CD4 epitope predictions in RV144: No strong evidence of a T-cell driven sieve effect in HIV-1 Breakthrough sequences from trial participants. PLoS One. 2014;9 doi: 10.1371/journal.pone.0111334 25350851 10.1371/journal.pone.0111334 PMC4211711

[46] Anderson JL, Fromentin R, Corbelli GM, Østergaard L, Ross AL. Progress towards an HIV cure: update from the 2014 International AIDS Society Symposium. AIDS Res Hum Retroviruses. Mary Ann Liebert, Inc.; 2015;31: 36–44. doi: 10.1089/AID.2014.0236 25257573 10.1089/aid.2014.0236 PMC4287112

[47] Li JZ, Etemad B, Ahmed H, Aga E, Bosch RJ, Mellors JW, et al The size of the expressed HIV reservoir predicts timing of viral rebound after treatment interruption. AIDS. NIH Public Access; 2016;30: 343–53. doi: 10.1097/QAD.0000000000000953 26588174 10.1097/QAD.0000000000000953 PMC4840470

[48] Li JZ, Etemad B, Ahmed H, Aga E, Bosch RJ, Mellors JW, et al The Size of the Expressed HIV Reservoir Predicts Timing of Viral Rebound after Treatment Interruption HHS Public Access. AIDS January. 28: 343–353. doi: 10.1097/QAD.0000000000000953 10.1097/QAD.0000000000000953 PMC4840470 26588174

[49] Williams JP, Hurst J, Stöhr W, Robinson N, Brown H, Fisher M, et al HIV-1 DNA predicts disease progression and post-treatment virological control. Elife. 2014;3: e03821 doi: 10.7554/eLife.03821 25217531 10.7554/eLife.03821 PMC4199415

[50] Piketty C, Lanoy E, Trabelsi S, Girard P, Tubiana R, Abramowitz L, et al. Persistence of anal squamous intraepithelial lesions and anal HPV infection in HIV-infected patientsdespite immune restoration under cART. Proc 12-th Int Conf Malig AIDS Other Acquir Immunodefic (ICMAOI); Infect Agents Cancer. 2010;5: 1–2. 10.1186/1750-9378-5-S1-A59

[51] Treasure GC, Aga E, Bosch RJ, Mellors JW, Kuritzkes DR, Para M, et al Relationship Among Viral Load Outcomes in HIV Treatment Interruption Trials. 2016;72: 310–313. doi: 10.1097/QAI.0000000000000964 26910502 10.1097/QAI.0000000000000964 PMC4911279

[52] Luzuriaga K, Gay H, Ziemniak C, Sanborn KB, Somasundaran M, Rainwater-Lovett K, et al Viremic relapse after HIV-1 remission in a perinatally infected child. N Engl J Med. NIH Public Access; 2015;372: 786–8. doi: 10.1056/NEJMc1413931 25693029 10.1056/NEJMc1413931 PMC4440331

[53] Henrich TJ, Hanhauser E, Marty FM, Sirignano MN, Keating S, Lee T-H, et al Antiretroviral-free HIV-1 remission and viral rebound after allogeneic stem cell transplantation: report of 2 cases. Ann Intern Med. NIH Public Access; 2014;161: 319–27. doi: 10.7326/M14-1027 25047577 10.7326/M14-1027 PMC4236912

[54] Shan L, Deng K, Shroff NS, Durand CM, Rabi SA, Yang HC, et al Stimulation of HIV-1-Specific Cytolytic T Lymphocytes Facilitates Elimination of Latent Viral Reservoir after Virus Reactivation. Immunity. 2012;36: 491–501. doi: 10.1016/j.immuni.2012.01.014 22406268 10.1016/j.immuni.2012.01.014 PMC3501645

[55] Borducchi EN, Cabral C, Stephenson KE, Liu J, Abbink P, Ng’ang’a D, et al Ad26/MVA therapeutic vaccination with TLR7 stimulation in SIV-infected rhesus monkeys. Nature. Nature Research; 2016;540: 284–287. doi: 10.1038/nature20583 27841870 10.1038/nature20583 PMC5145754

